# Autophagy and Metabolism in Normal and Malignant Hematopoiesis

**DOI:** 10.3390/ijms22168540

**Published:** 2021-08-09

**Authors:** Ioanna E. Stergiou, Efstathia K. Kapsogeorgou

**Affiliations:** Department of Pathophysiology, School of Medicine, National and Kapodistrian University of Athens, 11527 Athens, Greece; stergiouioanna@hotmail.com

**Keywords:** autophagy, mitophagy, metabolism, hematopoiesis, hematopoietic stem cells, leukemia

## Abstract

The hematopoietic system relies on regulation of both metabolism and autophagy to maintain its homeostasis, ensuring the self-renewal and multipotent differentiation potential of hematopoietic stem cells (HSCs). HSCs display a distinct metabolic profile from that of their differentiated progeny, while metabolic rewiring from glycolysis to oxidative phosphorylation (OXPHOS) has been shown to be crucial for effective hematopoietic differentiation. Autophagy-mediated regulation of metabolism modulates the distinct characteristics of quiescent and differentiating hematopoietic cells. In particular, mitophagy determines the cellular mitochondrial content, thus modifying the level of OXPHOS at the different differentiation stages of hematopoietic cells, while, at the same time, it ensures the building blocks and energy for differentiation. Aberrations in both the metabolic status and regulation of the autophagic machinery are implicated in the development of hematologic malignancies, especially in leukemogenesis. In this review, we aim to investigate the role of metabolism and autophagy, as well as their interconnections, in normal and malignant hematopoiesis.

## 1. Introduction

Hematopoiesis is a stepwise process through which hematopoietic stem cells (HSCs) differentiate to progenitor cells that demonstrate a restricted potential and eventually further differentiate to form all mature blood and immune cells. Effective hematopoiesis depends on the strict regulation of quiescence, self-renewal, and differentiation of HSCs. Healthy HSCs rely heavily on bone marrow oxygen tension to retain their stemness, and they do so by altering their gene expression to fit a more glycolytic rather than mitochondrial metabolic profile [1,2]. Differentiation commitment is reflected by a metabolic switch from Warburg glycolysis to the mitochondrial tricarboxylic acid cycle (TCA) and oxidative phosphorylation (OXPHOS) [1,2]. Malignant cells tend to rewire their metabolism to meet up with their high energy requirements for growth and proliferation. Metabolic reprogramming serves two main goals: first, rapid adenosine triphosphate (ATP) production and adequate supply of intermediates for the synthesis of nucleotides, amino acids, lipids, and redox molecules; second, the rewiring of nutrient sensing pathways [3]. Metabolic pathway aberrations, including glycolysis, the TCA cycle, and fatty acid metabolism, have been implicated in hematologic malignancies, especially leukemia [4]. 

Autophagy is the major intracellular degradation system by which cytoplasmic components are delivered to and degraded in the lysosome. Double-membrane vesicles, termed autophagosomes, engulf long-lived proteins or damaged organelles and transport these cargos to the lysosomes. There, the outer membrane of the autophagosome fuses with the lysosomal membrane, and the inner vesicle, together with its cargo, is degraded. The resulting macromolecules can be recycled back to the cytosol. The autophagic process serves a dual role: first, removal of damaged intracellular organelles; second, recycling of cellular contents, to support metabolites and basic building blocks required in anabolic processes, such as cell growth, proliferation, remodeling, and differentiation [5]. Autophagy has been shown to be essential for HSCs maintenance [6], while transcription factors, considered to be master regulators of hematopoiesis, exert their transcriptional control on autophagy-related genes [7,8]. Autophagy works to satisfy distinct metabolic demands during HSCs differentiation. The cellular mitochondrial content, as well as mitochondrial quality, defines the adequacy of OXPHOS, and thus ATP production, but, at the same time, the reactive oxygen species (ROS) levels. Mitophagy regulation during normal hematopoiesis serves the different metabolic requirements of hematopoietic cells from the HSC stage to mature blood cells and also controls the degree of DNA damage, through ROS modulation. Distinct levels of mitochondrial content and activity are a prerequisite for the maintenance and differentiation of hematopoietic stem/progenitor cells (HSPCs); hence, an optimal amount of mitophagy is critical. Hyperactivated mitophagy results in diminished mitochondrial activity, resulting in various hematopoietic disorders, such as aplastic anemia, hematological malignancies, and aging-associated diseases. Though research in leukemogenesis concentrates on recurrent genetic and epigenetic aberrations [9], recent data unravel that epigenome modulations contribute to the metabolic reprogramming of leukemic cells, while metabolites may conversely regulate epigenetic events [10]. This review focuses on the role of autophagy and metabolism during normal hematopoiesis, as well as on their perturbations during malignant hematopoiesis, with a focus on leukemogenesis.

## 2. The Role of Autophagy in Normal Hematopoiesis

### 2.1. Autophagy and HSCs

HSCs are unique cells capable of self-renewal through symmetric division and multi-lineage differentiation. They consist the pool that gives rise to both myeloid and lymphoid cells throughout the lifetime and are characterized by the ability to reconstitute the whole blood system of the recipient upon transplantation [11]. HSCs are generally characterized by being in a quiescent state, but upon specific stimuli, they self-renew and/or differentiate into hematopoietic progenitor cells (HPCs) that, in turn, give rise to mature hematopoietic cells [12]. Evidence supports that autophagy may play a role in the regulation of quiescence, self-renewal, and differentiation of HSCs [13,14]. 

Data supporting the notion that autophagy is an essential regulator of HSCs maintenance have initially been obtained from mouse models. Mortensen et al. demonstrated that conditional deletion of the essential autophagy gene *Atg7* in the hematopoietic system resulted in impaired self-renewal capacity, both in vitro (failure to form secondary colonies in colony-forming cell assays) and in vivo (repopulation assays). HSCs within the Lin(-) Sca-1(+) c-Kit(+) (LSK) compartment were significantly reduced, while *Atg7*-deficient LSK cells failed to reconstitute hematopoiesis in lethally irradiated mice. Autophagy impairment had a negative impact on the differentiation of HSCs into mature white and red blood cells, with *Atg7*-deficient mice displaying reduced myeloid and lymphoid progenitors, as well as severe anemia. Furthermore, decreased absolute counts of T, B, and NK cells in the peripheral blood were observed in these mice.

FIP200 (200-kDa FAK-family interacting protein) plays important roles in mammalian autophagy. Conditional deletion of FIP200 in hematopoietic cells leads to perinatal lethality and severe anemia in mouse models. FIP200 is required for the maintenance and function of fetal HSCs, since FIP200-deficient HSCs were unable to reconstitute lethally irradiated recipients. FIP200 ablation increased the rate of HSC proliferation and myeloid expansion, which may account for the depletion of fetal HSCs [15].

It is well established that the transcription factor FOXO3A regulates the expression of genes involved in the autophagic machinery, namely, *LC3B*, *Atg12*, *Atg4b*, *Ulk2*, *Vps34*, and *Beclin-1* [16]. It has been shown that mouse HSCs robustly induce autophagy after ex vivo cytokine withdrawal and in vivo calorie restriction, with FOXO3A being a key player in maintaining a gene expression program that ensures rapid autophagy induction upon starvation [7]. Salemi et al. demonstrated that in vitro cultured human HSCs show a high rate of autophagy, through which they probably ensure their capacity for self-renewal and differentiation. Inhibition of autophagy, either pharmacologically by 3-methyladenine or by ATG5 shRNA, resulted in HSCs’ failure to form colonies in in vitro colony-forming assays and to differentiate to neutrophils [17].

Mitophagy is a form of autophagy specific to the elimination of defective mitochondria. Normal modulation of the level of mitophagy regulates the maintenance of HSCs. One of the key regulators of mitophagy is the phosphatase and tensin homolog (PTEN)-induced putative kinase Pink1. Ito et al. showed that silencing of *Pink1* or *Parkin*, which encodes an E3 ubiquitin ligase necessary for Pink1 binding on the mitochondrial outer membrane and subsequent mitophagy induction, abrogates the ability of highly purified HSCs to expand in vitro but also affects their maintenance potential [18]. Mitophagy is generally considered a mechanism contributing to the maintenance of normal HSCs function. Excessive mitophagy, though, seems to be detrimental to HSCs maintenance and appears to block commitment to the hematopoietic lineage at the progenitor stage. Jin et al. identified the AAA+ -ATPase Atad3a as a regulator of Pink1-dependent mitophagy. Mice with hematopoietic system-specific deficiency in Atad3a exhibited hyperactivated mitophagy that resulted in aberrant hematopoietic homeostasis [19].

### 2.2. Autophagy and Erythropoiesis

Depletion of membrane organelles and extrusion of the nucleus are hallmarks of mammalian erythropoiesis. Betin et al. showed that autophagy is induced at the polychromatic erythroid stage, and that autophagosomes remain abundant until enucleation [20]. This stimulation of autophagy coincides with the transcriptional upregulation of many autophagy genes, namely, all *Atg8* mammalian paralog family members and a subset of *Atg4* family members [20]. *Atg7* deletion in mice leads to the development of severe anemia, with erythroblasts accumulating damaged mitochondria with an altered membrane potential, a phenomenon that eventually leads to cell death [21]. 

GATA-1, the master regulator transcription factor of erythropoiesis, has been shown to activate the essential autophagy component microtubule-associated protein 1A/1B-light chain 3 (LC3), as well as other autophagy-related genes [8]. 

Programmed mitochondrial clearance via autophagy is a prerequisite for reticulocyte maturation. The proapoptotic factor NIX (BCL2/adenovirus E1B 19 kDa protein-interacting protein 3 ligand (BNIP3L)), a BH3-only protein of the outer mitochondrial membrane, is upregulated during terminal erythroid differentiation [22,23]. NIX induces mitochondrial membrane depolarization and subsequent recruitment of LC3B/Gamma-aminobutyric acid receptor-associated proteins (GABARAP) to mitochondria [23]. Schweers et al. demonstrated that NIX is necessary for mitochondrial clearance in reticulocytes, since its deletion in vivo impairs mitophagy and erythroid maturation, resulting in anemia with reticulocytosis [23]. However, other autophagic processes such as ribosome clearance are not affected by NIX deletion. Kundu et al. demonstrated that Unc-51-like kinase 1 (ULK1) plays a critical role in the autophagic removal both of mitochondria and ribosomes during terminal erythroid maturation, though ULK1 deficiency leads to impaired, but not completely abolished, mitochondrial clearance [24]. Although Atg5 and Atg7 are indispensable for macroautophagy, their role in mitochondrial clearance remains controversial. It has been shown that mammalian cells use conventional Atg5/Atg7-dependent macroautophagy as well as an alternative ULK1-dependent Atg5/Atg7-independent macroautophagy process [25]. Honda et al. showed that mitochondrial clearance in fetal reticulocytes is ULK-1-dependent and Atg-5-independent, while ULK1-dependent macroautophagy was less involved in steady-state adult reticulocytes [26]. 

GATA-1, a transcription factor considered to be a master regulator of normal erythropoiesis, is implicated in the transcriptional regulation of mitophagy during erythrocyte maturation. GATA-1 transcriptionally controls and upregulates the expression of autophagy genes, namely, *Atg8* homologs, *Atg4b*, and *Atg12*, as well as lysosomal genes, namely, *Atp6v0e*, *Clcn7*, *Neu1*, and *Lamp1*, in primary human erythroblasts [8]. NIX expression is also upregulated by GATA-1 in primary human erythroblasts, thus rendering GATA-1 a regulator of mitophagy during erythroid maturation [23]. The KRAB/KAP1-miRNA regulatory cascade negatively regulates the miRNA-351 which targets NIX, thus increasing mitophagy during erythrocyte maturation. In mouse models, Kap1 deletion leads to reticulocytosis, mitochondrial retention, and fatal anemia [27]. Nuclear factor-erythroid 2 (NF-E2), another transcription factor crucial for normal erythroid maturation, upregulates the expression of BNIPL1 and ULK1, thus regulating mitophagy [28].

### 2.3. Autophagy and Granulopoiesis

*Atg7* deletion at the granulocyte-monocyte progenitor (GMP) stage inhibits neutrophil differentiation, resulting in accumulation of immature precursors. Impaired lipophagy was identified as responsible for the neutrophil differentiation defect upon *Atg7* deletion [29]. Moreover, autophagy-deficient mice with *Atg5* or *Atg7* deletion demonstrate impaired neutrophil function with defective nicotinamide adenine dinucleotide phosphate (NADPH) oxidase-mediated ROS generation and impaired neutrophil degranulation [30]. It has also been shown that the secretion of the proinflammatory cytokine IL-1β by neutrophils is mediated by autophagy [31].

### 2.4. Autophagy and Megakaryopoiesis

Megakaryocytic maturation, similar to erythroid maturation, is also regulated by the transcription factor GATA-1, which induces the expression of several autophagic genes [8]. Colosetti et al. used the chronic myeloid leukemia (CML) cell line K562 to prove that autophagy induction is an important event for megakaryocytic differentiation and is also accompanied by increased expression of Beclin 1 and p62/sequestosome 1 (SQSTM1) [32]. Using an *Atg7* hematopoietic conditional knockout mouse model, Cao et al. demonstrated that loss of autophagy resulted in the accumulation of mitochondria and ROS, impeding megakaryopoiesis and megakaryocyte differentiation, as well as thrombopoiesis, leading to abnormal platelets, larger in size and fewer in number, with deficient functions of activation and aggregation [33]. Disruption of the autophagic flux, by means of pharmacological inhibition, leads to impairment of platelet aggregation and adhesion in vitro, while *Becn1* heterozygous knockout mice display a prolonged bleeding time and reduced platelet aggregation [34]. Zhang et al. demonstrated that hypoxia induces mitophagy in platelets in a FUNDC1-dependent manner, regulating mitochondrial quality and quantity and controlling platelet activation during ischemia/reperfusion injury [35].

**Figure 1:**
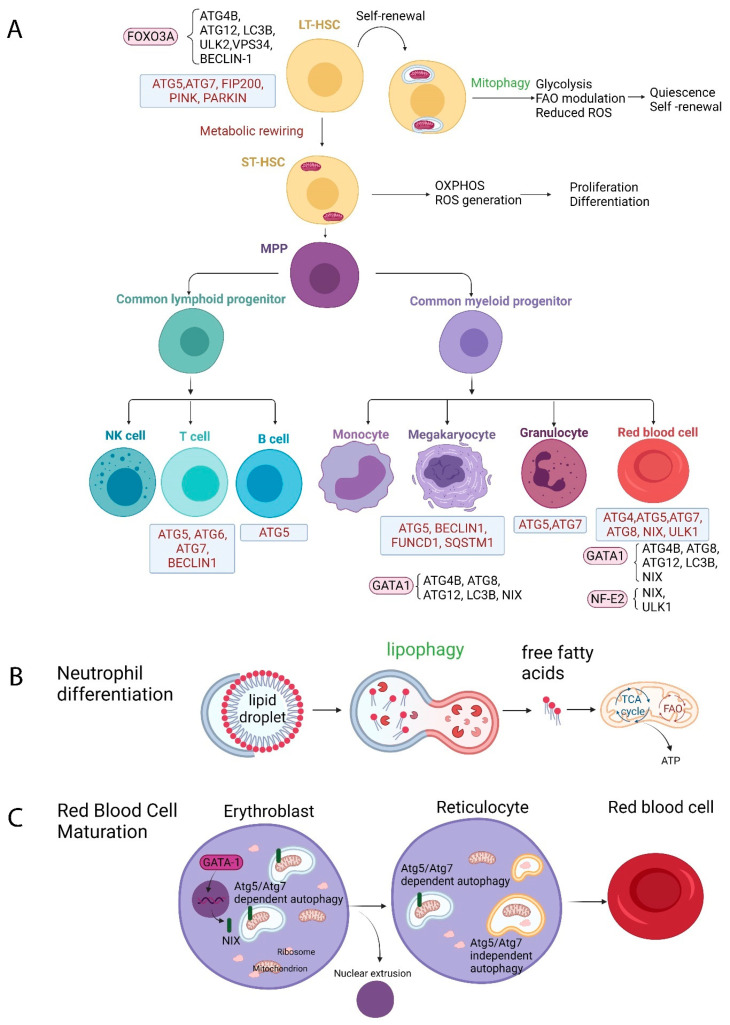
Overview of autophagy during normal hematopoiesis and its implications in metabolic reprogramming. (**A**) Components of the autophagic machinery required for normal hematopoiesis at different differentiation stages are shown in blue rectangular boxes, while transcription factors (FOXO3A, GATA1, NF-E2) regulators of hematopoiesis that control autophagic gene expression are shown in pink oval boxes. Long-term hematopoietic stem cells (LT-HSCs) maintain their self-renewal capacity and quiescence via mitophagy induction, which ensures a glycolytic metabolic profile and low reactive oxygen species (ROS) production, thus limiting DNA damage. Upon differentiation commitment, short-term hematopoietic stem cells (ST-HSCs) acquire a different metabolic profile, relying on oxidative phosphorylation (OXPHOS) to meet up with their increased energy requirements. (**B**) Lipophagy during neutrophil differentiation. Lipophagy delivers free fatty acids to the mitochondria, ensuring adequate substrates for the tricarboxylic acid cycle (TCA) and fatty acid oxidation (FAO) for the adenosine triphosphate (ATP) production needed during neutrophil differentiation. (**C**) Autophagy during terminal erythroid maturation. The proapoptotic factor NIX is necessary for mitochondrial clearance in reticulocytes, while it does not affect ribosome clearance. NIX expression is upregulated by the master regulator of erythropoiesis transcription factor GATA-1. ULK1 plays a critical role in the autophagic removal both of mitochondria and ribosomes during terminal erythroid maturation. FAO: fatty acid oxidation, LT-HSCs: long-term hematopoietic stem cells, MPP: multipotent progenitor, OXPHOS: oxidative phosphorylation, ROS: reactive oxygen species, ST-HSCs: short-term hematopoietic stem cells, TCA: tricarboxylic acid. Created with BioRender.com.

An overview of autophagy in normal hematopoiesis is described in Figure 1.

### 2.5. Autophagy and Lymphopoiesis

#### 2.5.1. T Lymphocytes 

*Atg5* or *Atg7* deletion in murine T cells leads to a decrease in thymocyte and peripheral T cell numbers, with *Atg5*-deficient T cells demonstrating decreased cell survival [36,37,38]. Stephenson et al. showed that *Atg5* deletion resulted in increased mitochondrial mass in peripheral T cells, while they also observed a correlation between mitochondrial mass and Annexin-V staining, proposing that autophagy is critical for mitochondrial maintenance and T cell survival [37]. Accordingly, Pua et al. showed that autophagy-deficient mature T cells fail to reduce their mitochondrial content in vivo and have increased ROS production, as well as an imbalance in pro- and antiapoptotic protein expression [38]. Recent findings highlight how the activation of transient receptor potential vanilloid 1 (TRPV1) induces autophagy in mice thymocytes through the ROS-regulated 5’ adenosine monophosphate-activated protein kinase (AMPK) pathway. TRPV1-triggered autophagy is Atg6/Beclin-1-dependent and involves ROS- and AMPK-mediated upregulation of Beclin-1 expression. Autophagy seems to be activated as a pro-survival process, as its inhibition triggers the apoptosis of thymocytes [39]. Moreover, Beclin-1-deficient mice display a dramatic reduction in thymic cellularity, since Beclin-1 expression is necessary for the maintenance of undifferentiated/early lymphocyte progenitor populations [40]. 

#### 2.5.2. B Lymphocytes 

Autophagy has been shown to be differentially required at different stages of B cell development. *Atg5* deletion in B lymphocyte progenitors results in a significant defect in B cell development at the pro- to pre-B cell transition, which is associated with increased cell death, indicating that Atg5 is important for B cell survival. Moreover, while B-1 B cells require Atg5 for their maintenance in the periphery, B-2 B cells are not affected by *Atg5* deletion [41]. Autophagy has also been shown to be implicated in the processes of plasma cell differentiation and in the maintenance of memory B cells [42,43].

## 3. Autophagy in Malignant Hematopoiesis

### 3.1. Autophagy in Acute Myeloid Leukemia (AML)

AML is a clonal hematopoietic disorder originating from leukemic stem cells (LSCs), which are responsible for disease initiation, chemotherapy resistance or failure, and disease relapse. LSCs originate from transformed HSCs or HPCs [44].

As it has already been analyzed, autophagy gene expression regulates HSCs maintenance. Deletion of the autophagy gene *Atg7* in HSCs resulted in pre-leukemic myeloproliferation in a mouse model and impaired production of both myeloid and lymphoid progenitors, while HSPCs displayed accumulation of mitochondria and ROS, as well as increased proliferation and DNA damage [45]. The loss of *Atg5* results in a similar myeloproliferative pre-leukemic phenotype to that described in mouse HSPCs models with *Atg7* loss, confirming the role of autophagy in HSPCs regulation [46]. 

Heterozygous deletions, missense mutations, or copy number changes in key autophagy genes have been identified with high frequency in AML patients, particularly those with complex karyotypes [46,47,48]. Jin at al. demonstrated that human AML blasts show lower transcript levels of *ULK1*, *FIP200*, *Atg14*, *Atg5*, *Atg7*, *Atg3*, *Atg4b*, and *Atg4d* compared to healthy donor granulocytes [49]. Autophagy-related genes *Atg10* and *Atg12*, *GABARAPL2* and *MAP1LC3B*, and *GABARAP* are, respectively, located at chromosome regions 5q, 16q, and 17p. Heterozygous chromosomal losses in these regions are frequently detected in AML patients, thus leading to decreased autophagic protein expression [46].

The reduced expression of autophagy genes found in human AML blasts and the subsequent decreased autophagic flux lead to accumulation of unhealthy mitochondria, implying that deficient autophagy may be beneficial for human AML. More importantly, heterozygous loss of autophagy in a mixed lineage leukemia-eleven nineteen (MLL-ENL) model of AML favored proliferation in vitro, and enhanced glycolysis along with leukemia aggressiveness in vivo [46]. Another study supporting the beneficial role of autophagy in AML comes from Sumitomo et al., who showed that inactivation of autophagy by *Atg5* or *Atg7* deletion prolonged survival in leukemic mice and reduced functional leukemia-initiating cells (LICs) [50]. *Atg7*-deficient LICs displayed enhanced mitochondrial activity and increased ROS production along with increased cell death. In addition, *Atg7* deletion markedly decreased leukemic cells in the peripheral blood, a phenomenon attributed to increased apoptosis, suggesting a higher dependency on autophagy compared to bone marrow leukemia cells. Finally, the authors suggested that autophagy may contribute to chemotherapy resistance, since cytarabine (AraC) treatment activated autophagy in LICs, and *Atg7* deletion potentiated the therapeutic effects of AraC, which included decreased LICs and prolonged survival [50]. Therefore, a reduced autophagic flux may provide an advantage for leukemogenesis.

However, Folkerts et al. demonstrated that autophagy levels were high in poor-risk AML, compared to favorable- and intermediate-risk AML [51]. Upon subdividing the CD34+ AML cells into ROS-low and ROS-high fractions, the authors showed that the AML CD34+ ROS-low cells exhibited higher levels of basal autophagy and decreased survival when treated with the inhibitor of autophagic lysosomal fusion, hydroxychloroquine [51]. Moreover, *Atg5* knockdown inhibited the in vivo maintenance of AML CD34+ cells in NOD scid gamma (NSG) mice [51]. This observation is in contrast to the previous published literature and supports a favorable role of autophagy in tumor proliferation.

Reports from MLL-AF9 AML, the most common alteration in infant AML, support that ATG5 or ATG7 is necessary for the initiation of AML, but once AML is established, autophagy is no longer required for the disease maintenance [52,53,54,55]. In an MLL-AF9-induced murine AML model, Saito et al. demonstrated that deletion of AMPK, a positive regulator of autophagy, significantly delayed leukemogenesis by increasing oxidative stress and DNA damage in LSCs [56]. LSCs overexpress FIS1, a regulator of mitochondrial dynamics that mediates mitophagy activity. Depletion of FIS1 attenuates mitophagy and leads to a profound loss of the LSCs self-renewal potential [57].

Nguyen et al. showed that loss of the selective autophagy receptor p62 impaired the expansion and colony-forming ability of leukemia cells, through mitophagy impairment and accumulation of defective mitochondria with subsequent increased ROS production, while it prolonged the latency of leukemia development in mouse models in vivo [58].

Rudat et al. showed that mechanistic target of rapamycin complex 1 (mTORC1)-mediated autophagy suppression stabilizes mutant Fms Related Receptor Tyrosine Kinase 3 (FLT3) in AML, while receptor tyrosine kinase inhibition increases autophagy and leads to FLT3 depletion [59]. On the contrary, Heydt at al. demonstrated that FLT3-internal tandem duplication (ITD) expression increases basal autophagy in AML cells, with the transcription factor ATF4 being identified as a key player in FLT3-ITD-induced autophagy [60]. ATF4 downregulation inhibited autophagy-dependent AML cell proliferation [60].

### 3.2. Autophagy in Myelodysplastic Syndromes (MDS)

Aberrations in the autophagic machinery have also been implicated in the pathophysiology of bone marrow failure syndromes. MDS comprise a heterogenous group of clonal hematopoietic disorders characterized by ineffective hematopoiesis and an increased risk of transformation to AML [61]. Mutations in autophagy genes are enriched in MDS and prevalent in higher-risk MDS patients [62].

In a mouse model, *Atg7* knockout in HSCs led to accumulation of mitochondria with an altered mitochondrial membrane potential in erythrocytes, causing cell death and anemia, similar to the MDS phenotype [21]. Erythroid precursors from patients with low-risk MDS demonstrate ultrastructural features of enhanced mitophagy [63], while erythroblasts from patients with high-risk MDS are characterized by the accumulation of enlarged and abnormal mitochondria [64]. Our team demonstrated that the mitophagy of abnormal mitochondria and autophagic death are prominently featured in the MDS myeloid lineage [65].

By assessing the transcript levels of the mitophagy receptor BNIP3, Lazarini et al. demonstrated decreased BNIP3 expression in MDS compared to healthy controls [66]. In another study, Jiang et al. reported decreased autophagosome formation, as well as decreased expression of the mitophagy receptor NIX and the autophagy promotor genes *AMPK* and *ULK1*, in bone marrow nucleated red blood cells from patients with high-risk MDS compared to controls and low-risk MDS patients. The high-risk group demonstrated a higher mitochondrial content and ROS production, whereas the mitochondrial membrane potential was lower, indicating mitochondrial dysfunction [67].

### 3.3. Autophagy in Chronic Myeloid Leukemia (CML)

CML is a myeloproliferative neoplasm characterized by the chromosomal translocation t(9;22)(q34.1;q11.2), which results in the formation of the *BCR-ABL1* fusion gene, encoding the BCR-ABL1 kinase [61]. BCR-ABL, through phosphoinositide-3 kinase (PI3K)/serine-threonine protein kinase B (AKT)/FOXO4 signaling, transcriptionally upregulates activating transcription factor 5 (ATF5) expression, which, in turn, stimulates the transcription of mechanistic target of rapamycin (mTOR), resulting in autophagy suppression in vitro [68]. Altman et al. demonstrated that BCR-ABL-expressing cells have low basal levels of autophagy but are highly dependent on this process and rapidly undergo apoptosis upon disruption of autophagy in vitro. This dependence on autophagy was extended in vivo, as *Atg3* deletion also prevented BCR-ABL-mediated leukemogenesis in a cell transfer model [69]. In vivo studies of a murine model of CML demonstrated that long-term (LT)-HSCs isolated from leukemic mice have higher basal autophagy levels compared with non-leukemic LT-HSCs and more mature leukemic cells [70].

Targeted (namely, tyrosine kinase inhibitor (TKI) imatinib mesylate) or non-targeted treatment in CML cell lines or patient samples has been shown to induce an increased autophagic response [71,72], attributed to ROS generation, endoplasmic reticulum (ER) stress, and increased autophagic gene and protein expression [71,73,74]. This increase in autophagic flux results in the senescence rather than the apoptosis of CML cells, rendering them resistant to treatment [75]. ATG7 and ATG4B, both key enzymes in the LC3 conjugation cascade, have been shown to play a critical role in CML CD34+ cell survival, while their genetic inhibition resulted in increased apoptosis of CML stem and progenitor cells and sensitized them to TKI therapy [76,77]. Karvela et al. demonstrated that loss of ATG7 resulted in metabolic reprogramming of CML cells, with impaired glycolysis and a shift towards OXPHOS, leading to ROS accumulation that caused CML CD34+ cells to differentiate towards the erythroid lineage [77]. Liu et al. showed that the AHI-1-BCR-ABL-DNM2 complex regulates endocytosis and ROS generation, both of which drive autophagy induction in CML [73]. Ianniciello et al. demonstrated that autophagy is required for CML CD34+ cell commitment as they transition from hypoxia to normoxia, simulating the leukemic commitment as cells migrate away from the hypoxic bone marrow niche [78].

### 3.4. Autophagy in Acute Lymphoblastic Leukemia (ALL)

Translocation t(12;21)(p13;q22), resulting in the ETV6-RUNX1 fusion protein (also known as TEL-AML1), is present in 25% of pediatric patients with B cell precursor ALL [79]. The autophagy-initiating lipidase VPS34 demonstrates increased expression in ALL patients with the ETV6-RUNX1 translocation compared to normal HSPCs. The causal relationship between ETV6-RUNX1 and autophagy was proved by the fact that VPS34 induction is transcriptionally regulated by ETV6-RUNX1 and correlates with increased levels of autophagy [80].

The BCR-ABL fusion protein is detected in 25–30% of adult and in 2–10% of pediatric B-ALL cases, and its detection is associated with poor prognosis [81]. In a murine model, hematopoietic progenitor cells expressing the p185 form of BCR-ABL had low basal levels of autophagy but were highly dependent on this process and rapidly underwent apoptosis upon disruption of autophagy through Atg3 deletion or treatment with chemical autophagy inhibitors. This dependence on autophagy was extended in vivo, as Atg3 deletion also prevented BCR-ABL-mediated leukemogenesis in a cell transfer model [69]. We can therefore conclude that hematopoietic cells expressing the BCR-ABL kinase depend on autophagy for survival, as well as for leukemic transformation. 

A transcript variant of the Beclin 1 gene carrying a deletion of exon 11, which encoded a C-terminal truncation of the Beclin 1 isoform, has been identified in the ALL 697 cell line, while this truncated Beclin 1 isoform displayed a reduced activity in induction of autophagy by starvation, indicating that it might function as a dominant negative modulator of autophagy [82].

High mobility group box 1 (HMGB1) is a nuclear DNA-binding protein which functions as a damage-associated molecular pattern molecule (DAMP), and its expression has been found increased in childhood ALL. As a positive regulator of autophagy, intracellular HMGB1 interacts with Beclin 1 in leukemia cells, leading to autophagosome formation. Additionally, exogenous HMGB1 directly induces autophagy and cell survival in leukemia cells [83].

Glucocorticoids, used widely for the treatment of B-ALL, have been shown to activate autophagy, subsequently inducing cell death [84]. Obatoclax, a bcl-2 inhibitor, overcomes glucocorticoid resistance in B-ALL cells, inducing ATG5-dependent autophagy [85]. Everolimus, an mTORC1 inhibitor, induces autophagy in B-ALL cells, a phenomenon that is alleviated by Beclin-1 downregulation by the siRNA strategy [86]. Patients with B-ALL resistant to glucocorticoid therapy showed different patterns of autophagy gene expression (26 upregulated and 10 downregulated) compared to patients with glucocorticoid-sensitive B-ALL [87]. L-asparaginase, used for the treatment of ALL, hydrolyzes glutamine to glutamic acid and asparagine to aspartic acid, reducing aerobic metabolism [88]. Treatment with L-asparaginase results in autophagy induction by ALL cells to compensate for the metabolic stress, while the use of the autophagy inhibitor chloroquine increases the sensitivity of ALL to L-asparaginase [89]. 

Glucocorticoids decrease the cellular intake of glucose and glutamine, while they increase glutamine synthesis [90]. The decrease in nutrient intake leads to an increase in autophagic flux to catabolically compensate the nutrient deficiency. This catabolic process produces ammonia, which is used by glutamate-ammonia ligase for intracellular glutamine synthesis, allowing the cells to ensure their own glucose supply [90].

### 3.5. Autophagy in Chronic Lymphocytic Leukemia (CLL) and Lymphoma

Autophagy was shown to have a pro-survival role in CLL cells, since autophagy inhibition by RNA interference targeting key autophagy genes or by using chloroquine or 3-methyladenine in peripheral blood mononuclear cells (PBMCs) from CLL patients decreased CLL cell viability [91]. On the other hand, Gade et al. demonstrated that inhibition of death associated protein kinase 1 (DAPK1), an autophagy-associated gene, reduces autophagy and promotes CLL growth [92]. A positive prognostic marker in CLL is the deletion of 13q14. This chromosomal region contains micro-RNA-15 and -16, which target the antiapoptotic protein BCL-2, with 13q14 deletion leading to BCL-2 upregulation [93]. BCL-2 binds to and negatively regulates BECN1, with BCL2 upregulation leading to suppression of BECN1 and autophagy inhibition [94]. SLAMF1 expression constitutes another positive prognostic factor in CLL and is involved in autophagosome recruitment and autophagic flux activation [95]. The opposing effects on autophagy regulation by the above-mentioned positive prognostic markers reveal the heterogenous impact of molecular CLL aberrations on autophagy.

Germinal center B cell-like-derived (GCB) diffuse large B cell lymphoma (DLBCL) is characterized by BCL-2 overexpression [61]. BCL-2 binds directly to BECN1, thus inhibiting autophagy [96]. Patients with decreased BCL-2 levels show increased BECN1 expression, which correlates with a favorable clinical outcome [97]. Monoallelic deletion of BECN1 in mice has been shown to promote tumorigenesis [98]. Bertolo et al. demonstrated that constitutive repression of autophagy responses in BCL6-driven lymphomas may contribute to lymphomagenesis [99]. On the other hand, Li et al. demonstrated that Cullin4B (CULB4) (a scaffold protein of the CUL4B-RING E3 ubiquitin ligase complex, highly expressed in DLBCL) is implicated in the positive regulation of autophagy, thus contributing to DLBCL progression. CUL4B deletion leads to inhibition of proliferation, an effect that could be attributed to the pro-survival ability mediated by autophagy [100]. Pharmacological inhibition of autophagy or knockdown of LC3B, ATG5, or p62/SQSTM1 in DLBCL OCI-Ly1 cells inhibited cell proliferation and induced apoptosis in vitro [101]. These data demonstrate the complex mechanisms implicated in autophagy regulation in DLBCL. 

Autophagy-related genes were found to be upregulated in follicular lymphoma (FL) lymph node tissues compared to reactive B cells [102]. In the same study, FL samples showed significantly decreased levels of both p62 and LC3 compared with reactive lymphoproliferation and DLBCL, indicative of increased autophagy activity in FL [102]. Wang at al. demonstrated that mutations in the ATP6V1B2 subunit of the vacuolar-type H+-translocating ATPase (v-ATPase) found in FL activate autophagic flux and maintain mTOR in an active state [103]. 

In mantle cell lymphoma (MCL), the t(11;14) translocation leads to cyclin D1 overexpression and high cyclin dependent kinase 4 (CDK4) activity. Inhibition of cyclin D1/CDK4 activity significantly reduces proteasome inhibitor-mediated stabilization of the proapoptotic BH3-only protein NOXA, mainly driven by an autophagy-mediated proteolysis. Combined treatment with the proteasome inhibitor bortezomib and autophagy inhibitors enhances NOXA stability, leading to super-induction of the NOXA protein, contributing to the apoptotic effect of proteasome inhibitor treatment [104]. Zhang et al. demonstrated that transglutaminase TG2 and nuclear factor-κB (NF-κB) signaling coordinates the survival of MCL cells via IL6-mediated autophagy [105], while Chen et al. demonstrated that ROS-induced C-X-C motif chemokine receptor 4 (CXCR4)/stromal cell derived factor 1 (SDF-1) signaling stimulates autophagy in MCL cells, promoting their survival in the bone marrow microenvironment [106]. 

## 4. Metabolism in Normal Hematopoiesis

The maintenance of the mammalian HSC system depends on the self-renewal of quiescent LT-HSCs and subsequent generation of short-term (ST)-HSCs, multipotent progenitors (MPPs) that will, in turn, differentiate to mature myeloid or lymphoid lineage cells [107,108]. Cells generate ATP, the major currency for energy-consuming reactions, through central carbon metabolism, including glycolysis and mitochondrial OXPHOS. It has been demonstrated that LT-HSCs contain fewer mitochondria compared to MPPs, thus relying on glycolysis to meet their energy demands [109,110]. This metabolic profile protects LT-HSCS from oxidative stress, maintaining low endogenous ROS levels, since aberrant ROS generation could abrogate various stem cell properties including cell cycle quiescence, self-renewal, survival, and multi-lineage differentiation capacity [111,112].

### 4.1. Regulation of HSC Metabolism–Glycolysis Versus OXPHOS

**Figure 2:**
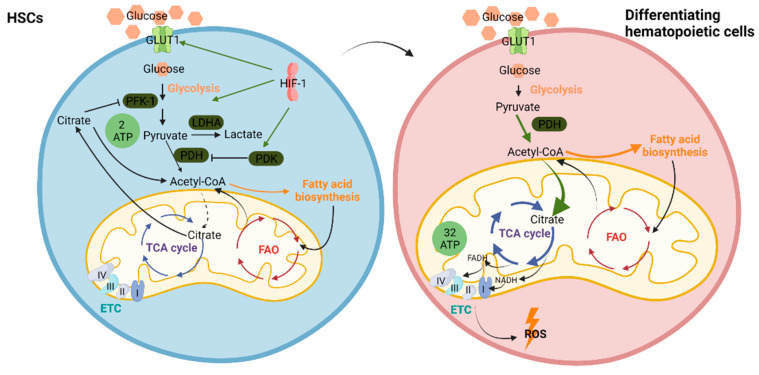
Metabolism in hematopoietic stem cells (HSCs) and differentiating hematopoietic cells. Under the transcriptional control of HIF-1, HSCs adopt a glycolytic metabolic profile that ensures low mitochondrial respiration and thus low ROS production. Differentiating hematopoietic cells depend on mitochondrial metabolism to ensure adequate energy supply for the process of differentiation. Acetyl-CoA: acetyl-coenzyme A, ETC: electron transport chain, FADH: flavin adenine dinucleotide, FAO: fatty acid oxidation, GLUT1: glucose transporter 1, HIF-1: hypoxia inducible factor 1, LDHA: lactate dehydrogenase A, NADH: nicotinamide adenine dinucleotide, PDH: pyruvate dehydrogenase, PDK: pyruvate dehydrogenase kinase, PFK-1: phosphofructokinase, ROS: reactive oxygen species, TCA: tricarboxylic acid. Created with BioRender.com.

Precise regulation of the levels of hypoxia inducible factor-1α (HIF-1α) ensures cell cycle quiescence of LT-HSCs that are maintained in a hypoxic state in the bone marrow niche [113,114]. The ability of pyruvate to enter the TCA cycle in the mitochondria is regulated by pyruvate dehydrogenase kinase-1 (PDK-1). PDK-1 expression has been shown to be under the transcriptional control of HIF-1α [115,116]. PDK-1 suppresses the activity of pyruvate dehydrogenase (PDH) and limits the influx of pyruvate into the TCA cycle. Suppression of pyruvate entry into the TCA cycle results in decreased citrate production. In growing cells, the citrate exported from mitochondria into the cytosol suppresses glycolysis at the level of phosphofructose kinase-1 (PFK-1), allowing a greater amount of glucose-6-phosphate to be diverted to nucleotide biosynthesis and glycosylation reactions. Furthermore, the cytosolic degradation of citrate provides the major source of acetyl-CoA for lipid and isoprenoid synthesis during cell growth. Therefore, HIF-1α-enhanced PDK-1 activity results in a decline in mitochondrial citrate production, leading to a derepression of PFK-1 activity and a concomitant suppression of lipid synthesis [117]. HIF-1α also controls the expression of lactate dehydrogenase A (LDH-A), an enzyme responsible for the production of lactate from pyruvate [118]. As LDH-A levels increase, cellular pyruvate is preferentially converted to lactate and secreted into the extracellular space [119]. Thus, under the transcriptional control of HIF-1α, the intracellular glucose utilization is diverted from lipid and nucleotide synthesis to ATP production via anaerobic glycolysis (Figure 2).

HIF-1α-dependent upregulation of PDK-1 in LT-HSCs represses the influx of glycolytic intermediates into mitochondria. This leads to an LT-HSC metabolic profile characterized by increased glycolysis levels compared to MPPs, necessary for the maintenance of cell cycle quiescence [120]. While LT-HSCs are characterized by a low mitochondrial potential and utilize glycolysis for ATP production, the differentiating hematopoietic cells rely on OXPHOS to meet their increased energy demands [110]. Pyruvate production during glycolysis generates only two molecules of ATP per molecule of glucose during anaerobic respiration, while upon entering the mitochondria to be used for OXPHOS, pyruvate generates 32 molecules of ATP per molecule of glucose (Figure 2). Although the ratio of ATP generation to glucose consumption under anaerobic glycolysis is inefficient compared to that generated by OXPHOS, the rate of ATP production under hypoxia potentially increases 100-fold compared to that supported by mitochondrial energy production under normoxia [121]. However, when dye-independent methods are used, namely, mitochondrial DNA quantification and enumeration of mitochondrial nucleotides, it has been shown that HSCs and MPPs have a higher mitochondrial mass than lineage-committed progenitors and mature cells. They present, though, a compromised respiratory and mitochondrial turnover capacity. Despite their similar mitochondrial mass, the respiratory capacity of MPPs exceeds that of HSCs. [122]. Luchinger at al. demonstrated that the mitochondrial protein mitofusin 2 (MFN2), which is implicated in mitochondrial fusion, is essential for the maintenance of HSCs with an extensive lymphoid potential [123]. Another study by Mohrin et al. showed that the mitochondrial unfolded protein response (UPRmt) is activated as HSCs transition from quiescence to the proliferation state [124]. Sirtuin 7 (SIRT7) is a component of the UPRmt. Its inactivation causes reduced quiescence, increased mitochondrial protein folding stress, and a compromised regenerative capacity of HSCs. SIRT7 expression is reduced in aged HSCs, and SIRT7 upregulation improves the regenerative capacity of aged HSCs [125].

A study by Ansó et al. demonstrated that loss of the mitochondrial complex III subunit Rieske iron–sulfur protein (RISP) in fetal mouse HSCs allows them to proliferate but impairs their differentiation, resulting in anemia and prenatal death, while RISP inactivation in adult HSCs also impaired respiration, resulting in loss of quiescence concomitant with severe pancytopenia and lethality. They, thus, concluded that mitochondrial respiration is dispensable for adult or fetal HSC proliferation, but essential for fetal HSC differentiation and maintenance of adult HSC quiescence [126].

The glycolytic phenotype of HSCs can be considered a protective mechanism that reduces ROS generation, which would cause oxidative stress and induce differentiation [127]. Mitochondria are the major source of ROS. However, HSCs rely on mitochondria when a metabolic switch is required. The transcription factor FOXO3 is implicated in the regulation of HSC metabolism. Studies show that FOXO3 exerts a protective effect on HSPCs regarding DNA damage, as FOXO3 deletion in HSCs alters mitochondrial function, leading to a deleterious ROS accumulation [128,129]. Tuberous sclerosis complex 1 (TSC1) is a negative regulator of mTOR, thus affecting the regulation of cellular metabolism [130]. Tsc1 deletion in HSCs results in a transition from quiescence to active cell cycling, with increased mitochondrial biogenesis and elevated levels of ROS, dramatically reducing both hematopoiesis and the self-renewal of HSCs [131]. Luo et al. demonstrated that mTOR overactivation contributes to HSCs’ premature exhaustion, in part, via induction of HSC senescence, while its inhibition promotes the ex vivo expansion and long-term hematopoietic reconstitution capacity of HSCs [132]. mTORC1 signaling is enhanced to promote the translation of mitochondria-associated transcripts during erythroid differentiation, controlling the mitochondrial content or erythroid lineage cells [133].

### 4.2. Fatty Acid Oxidation (FAO) in HSCs

FAO, another mitochondrial metabolic process, also plays a role in HSCs maintenance. Ito et al. identified a novel promyelocytic leukemia gene (PML)-peroxisome proliferator-activated receptor delta (PPARδ)-FAO pathway implicated in HSCs maintenance. Loss of PPARδ profoundly affects the maintenance of HSCs. Moreover, treatment with PPARδ agonists improves these HSCs functions, whereas, conversely, inhibition of mitochondrial FAO induces loss of the HSC compartment. Importantly, the authors demonstrated that PML exerts its essential role in HSCs maintenance through regulation of PPAR signaling and FAO. Mechanistically, the PML-PPARδ-FAO pathway controls HSC asymmetric division. Depletion of *Ppard* or *Pml*, as well as FAO inhibition, results in symmetric commitment of HSC daughter cells, while, conversely, PPARδ activation increases asymmetric division [134]. In line with these findings, Yu et al. showed that downregulation of the mitochondrial phosphatase protein tyrosine phosphatase 1 (PTPMT1) shifts the metabolic profile from glycolysis and FAO to mitochondrial OXPHOS, leading to ineffective hematopoiesis. This phenomenon is attributed to accumulating HSCs unable to differentiate due to increased entry of stem cells in the cell cycle followed by a pause in the G1 phase [135].

## 5. Metabolism in Malignant Hematopoiesis

### 5.1. Glucose Metabolism

It has been demonstrated that AML cell lines, as well as human primary AML blasts, show enhanced levels of glycolysis [136]. This glycolytic metabolic profile is regulated by the PI3K/AKT and mTOR pathways [137,138]. Published data indicate that increased glycolysis and a low efficiency of OXPHOS may contribute to drug resistance in AML. The expression of HIF-1α and GLUT1, as well as that of two of the key enzymes controlling the glycolytic flux, namely, hexokinase 2 (HK2) and LDH, was increased in bone marrow cells from AML patients with no remission (NR), compared to healthy control individuals and patients with complete remission (CR) and partial remission (PR). Studies indicate that highly glycolytic AML blasts are resistant to chemotherapeutic agents [136,139]. Herst et al. investigated the level of glycolytic metabolism in leukemic blasts as a prognostic marker in AML, demonstrating that highly glycolytic AML blasts were more resistant to apoptosis induced by all-trans-retinoic acid (ATRA) and/or arsenic trioxide in vitro, while high levels of glycolytic metabolism at diagnosis were predictive of a significantly improved duration of CR1 and overall survival following AML remission induction chemotherapy [136]. Chen et al., using a set of metabolite markers to create a prognostic model for AML, demonstrated that the low-prognostic risk score group showed enhanced glycolysis and an enhanced TCA cycle [139]. Evidence supporting the above results also comes from studies in mouse HSCs/HSPCs, where the deletion of LDHA and the M2 pyruvate kinase isoform (PKM2), both enzymes that regulate the last steps of glycolysis, inhibits leukemia initiation [140].

### 5.2. Glutamine Metabolism

Glutaminolysis ensures nitrogen supply for protein and nucleotide synthesis, while it also contributes to redox maintenance via NADPH production. Inhibition of glutamine uptake has been shown to induce apoptosis in AML cells, while treatment with L-asparaginase, an antitumor agent that also bears glutaminase activity, inhibits mTORC1 and protein synthesis in AML cells, thus producing a strong apoptotic response [141]. Stevens et al. showed that LSCs isolated from de novo AML patients rely on amino acid metabolism for OXPHOS and survival, with pharmacological inhibition of amino acid metabolism reducing OXPHOS and inducing cell death. On the contrary, LSCs obtained from relapsed AML patients are not reliant on amino acid metabolism due to their ability to compensate through increased fatty acid metabolism [142]. Gallipoli et al. identified glutaminase, the first enzyme in glutamine metabolism, as synthetically lethal with FLT3 inhibitor treatment in FLT3-ITD-mutated AML. They also demonstrated that glutamine metabolism, through its ability to support both mitochondrial function and cellular redox metabolism, becomes a metabolic dependency of FLT3-ITD AML, specifically unmasked by FLT3-TKI treatment [143]. Furthermore, it has been shown that PI3K/AKT signaling causes a metabolic switch from glutaminolysis to aerobic glycolysis in Notch-dependent T-ALL [144].

### 5.3. Fatty Acid Metabolism

Since LSCs use glutamine to support oxidative metabolism, their glutamine demands can be covered by fatty acid metabolism. Recent in vitro studies have shown that distinct genetic changes in AML are associated with enhanced dynamics and metabolism of lipid species in AML cells [145]. Isocitrate dehydrogenases (IDH1/2) are key metabolic enzymes, mutated in more than 15% of AML patients, with IDH1-mutant AML cells displaying a strongly reprogrammed lipid anabolism with dysregulation of fatty acid metabolism and fluxes [145].

Adipose tissue is one of the main sources of glutamine for cells. Bone marrow adipocytes from the tumor microenvironment support the survival and proliferation of malignant cells from patients with AML. AML blasts alter metabolic processes in adipocytes activating lipolysis, thus enabling the transfer of fatty acids from adipocytes to AML blasts [146]. Ye et al., using a mouse model of blast crisis CML, showed that adipose tissue provides leukemic cells with the necessary free fatty acids (FFA) to support their energy demands. High expression of the fatty acid transporter CD36 characterized the LSCs associated with adipose tissue [147]. Perea et al. also identified a CD36-expressing subpopulation within the CD34+ LSC compartment of human AML that was characterized by increased FFA uptake and FAO [148]. Studies from Tucci et al. demonstrated that ALL cells stimulate adipocyte lipolysis and utilize adipocyte-derived free fatty acids for proliferation, and this interaction may contribute to ALL resistance to chemotherapy [149,150]. A STAT-driven aberrant lipoprotein lipase (LPL) expression in CLL cells provides them with FFAs, supporting their oxidative metabolic capacity [151].

FAO is used by cancer cells to support their survival [152]. FAO generates acetyl-CoA that enters the TCA cycle, as well as flavin adenine dinucleotide (FADH2) and NADH that enter the ETC to generate ATP. It has been shown that carnitine palmitoyl transferase 1A (CPT1A) and carnitine transporter CT2, functioning in favor of FAO, are overexpressed in AML, with their inhibition compromising the growth and viability of AML cells [153,154]. Samudio et al. suggested that pharmacologic inhibition of FAO inhibited proliferation and sensitized human leukemia cells to apoptosis induction by ABT-737, a molecule that releases proapoptotic Bcl-2 proteins such as Bak from antiapoptotic family members [155]. 

Phospholipid metabolism alterations are frequently observed in cancer. Overexpression of choline kinases, responsible for the conversion of choline to phosphocholine (PC), results in increased PC levels that contribute to neoplastic transformation and cancer cell proliferation [156]. It has been demonstrated that PC levels in AML patients’ sera are higher in those with intermediate-risk AML compared to those with a favorable risk [4]. Lo Presti et al. demonstrated that deregulations in phospholipid metabolism can negatively affect prognosis, with PC and phosphoethanolamine (PE) being potential aggressiveness biomarkers [157]. A link between phospholipid metabolism and AML stemness has also been demonstrated, highlighting the important role of phosphatidylserine (PS) [158]. 

De novo fatty acid synthesis provides the membrane components required for the rapid proliferation of neoplastic cells, but it can also alter the physicochemical properties of the lipid bilayers, conferring with signaling and drug resistance. A lipidomic approach has been used for the stratification of AML patients, with t(8;21) and inv(16) patients (favorable-risk groups) presenting a substantial modulation of ceramide/sphingolipid synthesis compared to patients with normal karyotypes [159].

FAO was also shown to be implicated in lymphomagenesis. A subset of DLBCLs that do not require the activation of the B cell receptor for survival and demonstrate an OXPHOS gene expression signature catabolizes fatty acids at a higher rate than other subtypes of DLBCL and is highly dependent on FAO for survival and growth [160,161]. 

### 5.4. Mitochondrial Metabolism

Compared with normal hematopoietic cells, AML cells have a higher mitochondrial mass, without a concomitant increase in respiratory chain complex activity, thus displaying a lower spare reserve capacity in the respiratory chain that renders them more susceptible to oxidative stress [162]. Cytarabine-resistant AML cells display high levels of ROS, with an increased mitochondrial mass and active polarized mitochondria, findings consistent with a high OXPHOS status. Cytarabine residual cells exhibit increased FAO, upregulated CD36 expression, and a high OXPHOS gene signature, predictive for treatment response in patient-derived xenografts and patients with AML [163]. 

Both in normal and malignant hematopoiesis, the redox status is defined by the production of ROS and the function of antioxidant systems. ROS production depends on two major sources: the mitochondria, and the nicotinamide adenine dinucleotide phosphate oxidases (NOX) activity. NOX functional polymorphisms in de novo AML patients have been associated with response to induction therapy, with higher ROS generation leading to improved complete remission rates [164]. Constitutive activation of NOX contributes to extracellular ROS production in patients with AML, while mitochondrial ROS production is rarely observed [165]. These increased ROS levels were shown to promote AML blast proliferation [165]. Activating mutations of the FLT3 receptor are detected in approximately one third of newly diagnosed AML cases, with ITD mutations being associated with an adverse prognosis [166]. The increased levels of endogenous ROS demonstrated in FLT3-ITD-mutated AML could be associated with the aggressiveness and poor prognosis of these AML cases [167]. Moreover, it seems that chemo-resistant AML displays high levels of ROS, along with an active OXPHOS status [163]. Redox balance deregulation in de novo AML patients has been correlated with the molecular status and overall survival [168]. However, compared to normal hematopoietic cells, AML cells display a higher mitochondrial mass, though without increased respiratory chain complex activity, resulting in lower spare reserve capacity of the respiratory chain and increased susceptibility to oxidative stress [162]. It has been shown that NOX2-derived superoxide stimulates mitochondrial transfer from bone marrow stromal cells (BMSC) to AML blasts through AML-derived tunneling nanotubules [169].

LSCs are characterized by a distinct metabolic profile different from that of healthy HSCs. Lagadinou et al. demonstrated that quiescent LSCs are deficient in their ability to employ glycolysis and are highly reliant on OXPHOS while still maintaining low ROS. These ROS-low LSCs are unable to utilize glycolysis when mitochondrial respiration is inhibited, thus rendering the maintenance of mitochondrial function essential for LSC survival [170].

It has been shown that in MLL-AF9-induced murine AML, deletion of AMPK, a positive regulator of autophagy, significantly delays leukemogenesis and depletes LSCs by reducing the expression of glucose transporter 1 (Glut1), compromising glucose flux, and increasing oxidative stress and DNA damage [56]. FIS1 is essential for mitophagy activity in LSCs. Inhibition of AMPK, which lies upstream of FIS1, recapitulates the biological effect of FIS1 loss, attenuating mitophagy and leading to a decreased self-renewal potential in LSCs [57].

Increased ROS production leads to DNA damage and genomic instability, and it has, therefore, been implicated in bone marrow failure [171]. In mouse models, ablation of the proofreading activity of the mitochondrial DNA polymerase gamma (Polg) leads to accumulation of mitochondrial DNA mutations and dysfunctional mitochondria. These animals develop fatal megaloblastic anemia that is associated with both erythrodysplasia and impaired lymphopoiesis, findings frequently observed in human patients with MDS [172]. Mitochondrial DNA mutations have been found in patients with MDS [173,174,175]. Metabolic analyses of the MDS stem cell compartment show a profound activation of the protein synthesis machinery and increased oxidative phosphorylation [176]. Poulaki et al. demonstrated that myeloid bone marrow cells from MDS patients with low blast counts exhibit accumulation of glycolytic intermediates due to failing mitochondria, while their counterparts from MDS patients with high blast counts display a functional ETC and improved redox, compensating for the Warburg disruption [177]. 

Metabolic analyses on both stem cell-enriched (CD34+ and CD34+CD38-) and differentiated (CD34-) cells derived from individuals with CML indicate that primitive CML cells rely on upregulated oxidative metabolism for their survival [178]. Combination treatment with imatinib and tigecycline, an antibiotic that inhibits mitochondrial protein translation, selectively eradicates CML LSCs both in vitro and in a xenotransplantation model of human CML [178].

ALL cells may also have high levels of mitochondrial oxidative metabolism, since inhibition of mitochondrial respiration by tigecycline in primary lymphocytes and CD34 progenitors from ALL patients inhibits growth and induces apoptosis [179]. Zhong et al. reported that T cell acute lymphoblastic leukemia (T-ALL) cells are characterized by increased oxidative phosphorylation and robust ATP production. They demonstrate that oxysterol binding protein-related protein 4 (ORP4L) is expressed in T-ALL but not normal T cells, and its abundance is proportional to cellular ATP [180].

When studying therapy-naïve CLL patients, Jitschin et al. identified a metabolic profile characterized by increased oxidative stress, linked to alterations in the lymphoid compartment. Increased oxidative phosphorylation without increased aerobic glycolysis characterized the CLL cells. CLL cells adapted to intrinsic oxidative stress by upregulating the stress-responsive heme-oxygenase-1 (HO-1), thus implying that HO-1, besides its function as an antioxidant, may promote mitochondrial biogenesis [181].

A subset of DLBCLs that do not require the activation of the B cell receptor for survival has an OXPHOS gene expression signature, indicating that they are highly dependent on mitochondrial metabolism for survival [160,161].

**Figure 3:**
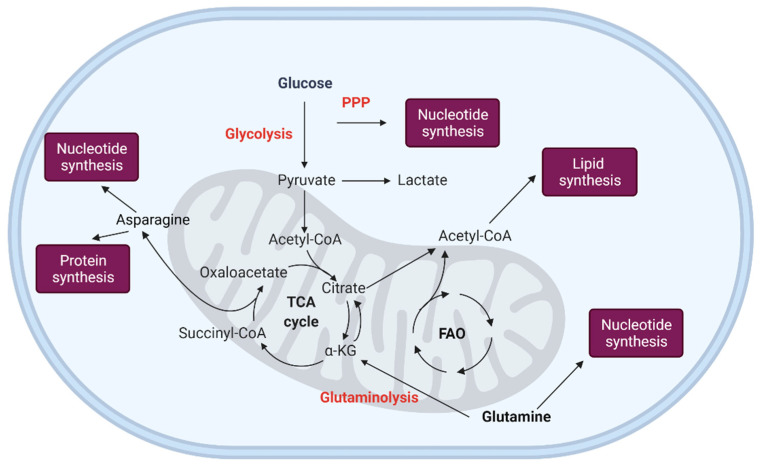
Leukemic stem cell metabolism. Leukemic stem cells demonstrate a glycolytic metabolic profile, with the branching from the upper glycolysis pentose phosphate pathway (PPP) contributing to nucleotide synthesis. Fatty acid oxidation (FAO) provides acetyl-CoA, which supports the TCA cycle and is also used for lipid synthesis. Glutaminolysis supports oxidative metabolism and supplies nitrogen for protein and nucleotide synthesis. Acetyl-CoA: acetyl-coenzyme A, FAO: fatty acid oxidation, PPP: pentose phosphate pathway, TCA: tricarboxylic acid. Created with BioRender.com.

Figure 3 summarizes the main pathways implicated in LSCs metabolism.

## 6. Links between Autophagy and Metabolism

Nutrient deprivation dramatically increases autophagosome formation, which, in turn, leads to the generation of amino acids, glucose, free fatty acids, and ATP, through autophagic degradation of metabolic macromolecules [182]. The highest levels of autophagy are induced by nitrogen or amino acid deprivation in yeast and cultured mammalian cells, respectively. This comes as no surprise given that the main products of autophagy are protein-derived amino acids. Once the cellular levels of amino acids are restored, the reactivation of the mTORC1 complex leads to autophagy termination [183]. During starvation, protein anabolism is generally downregulated, but it has been suggested that autophagy-derived amino acids are used for the synthesis of proteins essential for adaptation to starvation. These include lysosomal enzymes, respiratory chain proteins, antioxidant enzymes, and proteins implicated in the amino acid synthesis pathway [184,185]. Autophagy-defective yeast mutants, being unable to synthesize these proteins, lose their respiratory function and accumulate high levels of ROS, further decreasing their mitochondrial DNA content [185]. Autophagy can also contribute to ATP production, since autophagy-derived amino acids can be converted to TCA cycle intermediates. Guo et al. showed that autophagy maintains TCA cycle metabolites in Ras-expressing neoplastic cells [186]. Moreover, lipophagy, which is the degradation of lipid droplets by autophagy, also contributes to energy production [187].

Glucose metabolism, through glycolysis, leads to pyruvate formation, which is subsequently oxidized to generate ATP via the TCA cycle and mitochondrial OXPHOS. Insulin is known to stimulate glycolysis and lipogenesis and to suppress gluconeogenesis [188]. During insulin resistance, Sirtuin1, an NAD-dependent deacetylase, is downregulated [189]. Nutrient depletion leads to NAD+ accumulation at the expense of NADH and induces autophagy via Sirtuin1 activation [190]. Cancer cells use glucose as their major energy source, through metabolic pathways different from those used by non-cancer cells. During aerobic glycolysis, normal cells use glycolysis metabolites for ATP production through mitochondrial oxidative phosphorylation. The oxygen-deprived cancer microenvironment leads cancer cells to perform anaerobic glycolysis, during which glucose is transformed to lactate for ATP production. Cancer cells preferentially use aerobic glycolysis for ATP production even under sufficient oxygen supply, a phenomenon known as the Warburg effect [191]. HIF-1 is one of the major drivers of the Warburg metabolism adopted by cancer cells [192]. HIF-1 stabilization leads to the expression of glucose transporters and glycolytic enzymes, favoring the conversion of pyruvate to lactate over its incorporation into the TCA cycle [193]. Furthermore, HIF-1 also induces the pro-mitophagic receptors BNIP3 and ΝΙΧ, promoting mitophagy and therefore mitochondrial mass reduction, which results in reduced oxygen consumption [194]. During normal hematopoiesis, HIF-1 stabilization determines the glycolytic metabolic profile of LT-LSCs, necessary for the maintenance of cell cycle quiescence [120].

Autophagy has been identified as an essential protective mechanism of HSCs against metabolic stress [45]. Watson et al. demonstrated that healthy human and mouse HSPCs show an enhanced basal autophagic flux compared to more committed/mature hematopoietic cells, thus ensuring autophagic mitochondrial clearance and lower ROS levels [46]. Since mitochondria are a major source of ROS, mitophagy in HSCs acts a “quality control” mechanism. Elevated ROS levels may be deleterious for HSCs, since, after an initial hyperproliferative HSC state, they eventually lead to HSC exhaustion through apoptosis [195,196]. Healthy HSCs have a lower number of mitochondria compared to more differentiated hematopoietic cells, reflecting their dependency on glycolytic metabolism to meet their energy requirements, while, as differentiation proceeds, mitochondrial OXPHOS ensures an adequate energy supply to meet the increasing energy demands [110,197].

Impairment of autophagy in HSCs leads to an increased mitochondrial content. Both ATG7 and FIP200 deletions in mice HSCs resulted in an increased mitochondrial mass and upregulated ROS production compared to healthy HSCs [15,45]. Pereira et al. investigated how signaling pathways and autophagy are implicated in the metabolic regulation of AML cell lines. They demonstrated that constitutive coactivation of AMPK and mTORC1 and increased autophagy are associated with OXPHOS metabolism in AML cells, while activation of AKT/mTORC1 and low autophagy flux are associated with a glycolytic profile of AML cells [138]. Karvela et al. demonstrated that autophagy inhibition, via ATG7 knockdown, in Philadelphia chromosome-positive cells results in decreased glycolysis and increased carbon flux in the mitochondrial TCA cycle, leading to increased oxidative phosphorylation and mitochondrial ROS accumulation [77].

Mitochondrial biogenesis is regulated by peroxisome proliferator-activated receptor gamma coactivator-1α (PGC-1α) and c-Myc. Under hypoxia, HIF-1 downregulates PGC-1α to ensure engagement in glycolytic metabolism [198]. The MAPK pathway stabilizes PINK1 and promotes mitophagy in response to ROS-induced stress, leading to the clearance of damaged mitochondria [199]. Hyperactivated PINK1-dependent mitophagy in hematopoietic cells results in aberrant hematopoietic homeostasis [19]. Our team demonstrated that pseudohypoxic HIF-1 stabilization drives mitophagy in differentiating myeloid bone marrow cells from patients with MDS, thus contributing to ineffective hemopoiesis [65].

Mitochondrial biogenesis and mitophagy can generate a pool of mitochondria better suited to catabolize fatty acids via FAO [200]. FAO provides metabolic fuel for cancer cells when extra ATP is needed [152]. Fatty acid oxidation leads to increased lipid-derived acetyl-CoA, which works as a source of carbon for histone acetylation [201]. PGC1α could orchestrate the metabolic pathway of FAO, since it has been shown that its upregulation promotes FAO [202]. PGC1α may connect lipid metabolism, mitophagy, and mitochondrial homeostasis, since it is known to regulate mitophagy during myogenesis, buffering ROS-mediated removal of mitochondria [203]. Activation of the PPAR (peroxisome proliferator-activated receptor)–fatty acid oxidation pathway promotes the expansion of highly purified HSCs through enhanced Parkin recruitment in mitochondria, which drives mitophagy initiation [18]. During normal granulocyte differentiation, lysosomal breakdown of lipids supports the required substrates for mitochondrial FAO, thus contributing to the metabolic switch from glycolysis to mitochondrial OXPHOS. In maturing granulocytes, this switch is accompanied by a decrease in lipid droplets in an autophagy-dependent manner. Riffelmacher at al. demonstrated that Atg7 deletion in granulocyte precursors leads to lipid droplet accumulation, proving that this lipophagy defect hinders the required neutrophil maturation metabolic reprogramming to OXPHOS. This phenotype could be rescued in vitro by free fatty acid supplementation or pyruvate, an alternative to fatty acid-derived acetyl-CoA for oxidative phosphorylation [29].

Table 1 summarizes autophagy aberrations implicated in myeloid leukemogenesis and their effects on the metabolism.

## 7. Conclusions

Autophagy and metabolism are highly conserved cellular functions that ensure the quiescence, self-renewal capacity, and differentiation potential of HSCs, as well as the normal maturation of differentiated hematopoietic cells. It is well established that autophagy is a key regulator of the metabolic rewiring during normal hematopoiesis. Aberrations in metabolic pathways and the autophagic machinery have been described in malignant hematopoiesis. Currently published studies indicate a controversial role of autophagy in leukemogenesis, with some identifying deficient autophagic machinery in the leukemic cell, and others supporting the notion of increased autophagic flux. There is no doubt, though, that dysregulated autophagy affects the metabolic profile of hematopoietic cells, contributing to ineffective hematopoiesis and hematopoietic malignancy, while it has also been shown to participate in responses to treatment, especially in the leukemia setting. Further research is definitely needed to unravel these interconnections between autophagy and metabolism in normal and malignant hematopoiesis, since their targeting may affect future therapeutic approaches.

## Figures and Tables

**Table 1:** Summary of autophagy aberrations implicated in myeloid leukemogenesis and effects on metabolism.

Autophagy Aberrations	Leukemia Model	Metabolic Effect	
ATG5 deletion	Myeloproliferation (mouse models)		[46,50]
ATG7 deletion	Myeloproliferation (mouse models)	Accumulation of mitochondria Increased ROS	[45,50]
Low transcript levels of ULK1, FIP200, ATG14, ATG7, ATG3, ATG4B, ATG4D	Human AML blasts		[49]
Loss of ATG10, ATG12, GABARAPL2, MAP1LC3B, GABARAP	AML with deletions of 5q, 16q, 17p		[46]
AMPK deletion (downregulation of autophagy)	MLL-AF9-induced murine AML model	Increased oxidative stress	[56]
FIS deletion (mitophagy attenuation)	Human LSCs	Reduced mitochondrial activity	[57]
Loss of p62 (mitophagy impairment)	Murine myeloid leukemia	Increased ROS	[58]
AKT/mTORC1 activation (downregulation of autophagy)	AML cell lines	Glycolysis enhancement	[138]
Downregulation of ATF4 (autophagy inhibition)	FLT3-ITD-mutated AML cell lines		[60]

## Data Availability

Data sharing not applicable.
